# Blood factory: which stem cells?

**DOI:** 10.1186/s12878-018-0105-4

**Published:** 2018-05-10

**Authors:** Maria Teresa Esposito

**Affiliations:** Department of Life Sciences, University of Roehampton, Whiteland College, London, SW15 4JD UK

**Keywords:** Stem cells, Blood transfusion, Red blood cells

## Abstract

Blood transfusions are often essential for treatment of severe anaemia and pregnancy complications. The unavailability of blood is a medical concern, especially in developing countries. New sources of red blood cells (RBC) are under investigation. Several studies have attempted to produce functional RBC from CD34+ haematopoietic stem cells (HSC) isolated from peripheral blood and umbilical cord blood, from embryonic stem cells (ESC) and induced pluripotent stem cells (iPSC). A recent article published in Nature Communications describes a novel model for generating RBC from a stable erythroid cell line obtained from bone marrow CD34+ haematopoietic stem cells (HSC). The cells generated by this method are phenotypically and functionally adult RBC, that resemble very well the donor RBC. In vivo experiments confirmed no difference in the survival of these RBC and donor RBC. The study therefore highlights that this immortalized line is a promising new source of adult RBC.

## Background

More than 90 million blood transfusions are carried out every year in the world. The demand for blood is high in both developed and developing countries. In developed countries with an improved lifespan, an aging population with a higher prevalence of diseases might explain the high demand for blood supply. In developing countries blood transfusions are essential for treatment of childhood anaemia and pregnancy complications. While in developed countries the blood demand might be adequately met by blood donors, who often represent 1–4% of population, in developing countries the blood supply is severely inadequate. Data from the World Health Organization (WHO) estimate that developing countries collect less than 10 donations per 1000 inhabitants. Although blood transfusions can be life-saving they are not without risks. Immunological complications, infections, iron overload and blood mismatch raise concerns and pose a serious risk to the patients requiring transfusions [1]. Therefore, the need of blood is equal important as the need of reducing risks for patients.

Stem cells represent an interesting alternative to blood transfusions as they could ensure adequate supplies of RBC of a particular blood group and reduce some of the risks associated with blood transfusions, such as infections.

**Fig. 1:**
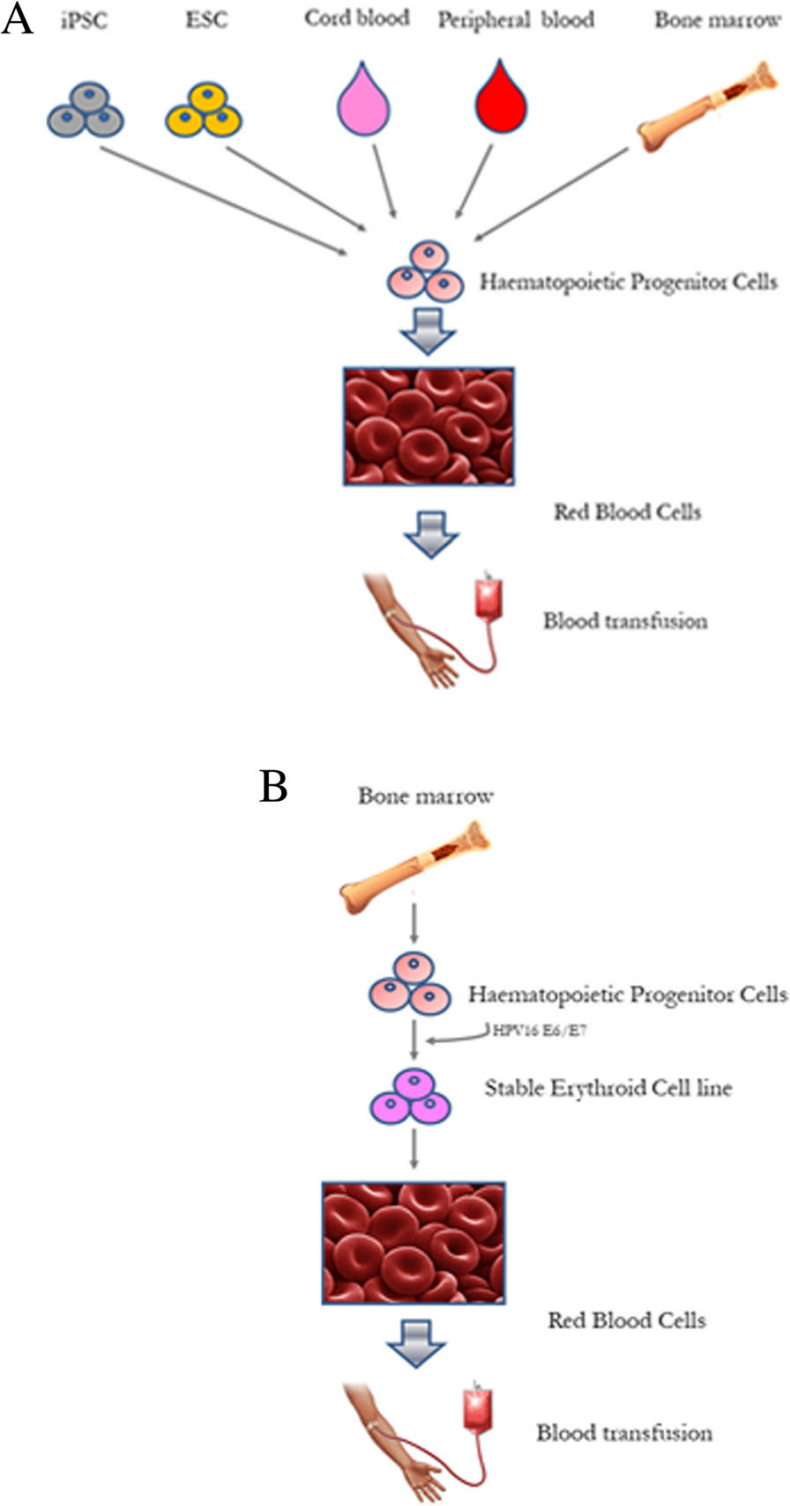
Schematic representation of Red Blood Cells (RBC) generation from distinct sources of stem cells. **a**) RBC can be derived from multiple sources of stem cells: iPSc, ESC, cell lines, cord blood, peripheral blood and bone marrow. **b**) BEL-A cell line was obtained by immortalization of bone marrow CD34+ HSC with HPV16-E6/E7

Red blood cells (RBC) can be produced from stem cells [2]. Several studies have attempted to produce functional RBC from CD34+ haematopoietic stem and progenitor cells (HSC) isolated from peripheral blood [3–5] and umbilical cord blood [3, 5–9], or from human embryonic stem cells (ESC) [5, 10–14] and induced pluripotent stem cells (iPSC) [15–17] utilizing different methods for the differentiation of the stem cells along the erythroid pathway (Fig. 1a).

The first attempts to generate functional RBC from CD34+ HSC isolated from peripheral and umbilical cord blood relied on co-culture with feeder layer to mimic the bone marrow microenvironment and support cell expansion and erythroid differentiation [3, 8]. Giarratana et al. compared the characteristics of RBC obtained from HSC of diverse origins and expanded with or without feeder layer [3]. Their results show that in absence of feeder layer, cell expansion and differentiation is not altered but the enucleation, which corresponds to terminal differentiation, is severely impaired indicating that the feeder layer provides fundamental signals for the complete maturation of RBC [3].

However, the complexity of a co-culture system with feeder layer impairs the scaling-up of this process for industrial development.

Years later, the same group and others published feeder-layer free protocols based on cocktails of cytokines that allowed the maturation of erythroid precursors into enucleated cells at a high rate [4–7].

Giarratana et al. generated RBC peripheral blood CD34+ HSC obtained by leukapheresis after mobilization with G-CSF and demonstrated for the first time that these cells could survive in a human patient as long as donor RBC [4].

The authors used a feeder-layer free protocol based on a cocktail of cytokines (SCF, IL3 and EPO) that ensured proliferation and erythroid differentiation of CD34+ HSC. The percentage of enucleated cells and adult haemoglobin were both very high (over 80%). Theoretically, one unit of peripheral blood or cord blood can yield multiple units of RBCs because the erythroid progenitors can be expanded in vitro. However, the expansion potential of the peripheral blood CD34+ progenitors is still a limiting factor, highlighting the difficulty in adapting this procedure to the generation of RBCs for routine clinical application.

Although peripheral HSC offer the unique advantage to allow the production of autologous RBC, some studies have reported that cord blood CD34+ HSC are a better sources of RBC because they have an increased proliferation potential [3, 5].

RBC generated from cord blood CD34+ HSC show high percentages of foetal haemoglobin, which has higher affinity to oxygen than adult haemoglobin. However, Neildez-Nguyen et al. observed that upon in vivo injection a switch from foetal to adult hemoglobin occurred, suggesting that complete maturation of the RBC took place in the bone marrow of the host mice [6].

An alternative solution to improve the yields is developing a stable source of cells that can be used for RBC production.

Human ESCs and iPSC give rise to erythroid progenitors that have an expansion potential which in vitro is greater than the potential of the progenitors derived from bone marrow, peripheral blood or cord blood. More importantly, these cells represent a limitless and immortal source of RBC [18].

In 2007 Lu et al. [11] obtained a precursor of haematopoietic and endothelial cells, called haemangioblast, from human ESC. The authors demonstrated the ability of this cell to differentiate along multiple haematopoietic lineages as well as into endothelial cells, in typical CFU assays. However, the CFU-erythroid cells obtained were very primitive. The erythrocytes were all nucleated and mainly expressed foetal globin genes [11]. A year later, in 2008 the same team published an improved protocol that led to an increase in yield and maturation of erythroid progenitors [12, 13]. By growing the cells onto a feeder layer the authors obtained enucleation in up to 65% of cells and the expression of *β*-globin in up to 15% of the cells [12]. The authors attempted also to generate blood groups A, B, O, and both Rhesus D positive and Rhesus D negative. However, the authors were unable to produce the O Rhesus D negative blood type, the so-called “universal” donor, because of the lack of availability of human ESC lines containing the O-negative gene [12]. Other studies demonstrated the generation of erythroid progenitors from ESC with enforced expression of transcription factors, such as *RUNX1* and *HOXB4* but these studies present limitations in the ability of the cells to survive in vivo and give rise to adult RBC [2, 10, 14]. Moreover, the use of transcription factor-overexpression and feeder layer as well as ethical concerns over the use of cells of embryonic origin represent major hurdles for further translation to clinical applications.

Since the breakthrough study of Shinya Yamanaka and the generation of iPSC cells [19] several attempts have been made to generate RBC from HSC derived by iPSC. This approach circumvents the ethical concerns of using embryonic stem cells, however it is limited by similar technical pitfalls, including transcription factors overexpression, use of viruses and feeder layer.

In 2010 Lapillonne et al. described for the first time a 2-step, feeder-free system to produce RBC from iPSC [15]. They generated iPSC from human foetal and adult fibroblasts using the method described by Thomson’s group [20]. However, by using this method, they obtained enucleation in only up to 10% of RBC generated from iPSC, and 66% of RBC generated from ESC. Even in this case the majority of the RBC expressed the fetal *γ*-globins [15]. Similar results were obtained by Feng et al. [16].

Therefore, although very attractive, ESC and iPSC might not represent the best choice of stem cells for blood supply. The differentiation protocols are very long and RBCs derived from ESCs and iPSC are not fully adult RBCs and maintain foetal characteristics. RBC derived from ESCs and iPSC express embryonic *ε*– and foetal *γ*-globins with low levels of detectable adult *β*-globin. Although ESC are superior to iPSC for the amplification and percentages of enucleated RBC, the enucleation, which represents an important step in the differentiation of an erythroid progenitor in a mature RBC, does not reach in iPSC-derived RBC the same percentages achieved with RBC obtained from cord and peripheral blood CD34+ HSC.

An alternative strategy is to generate RBC from immortalized adult erythroid progenitor cell lines. This strategy could circumvent the limitations of ESCs and iPSC providing a sustainable and reliable source of adult RBC (Fig. 1b).

A recent study authored by Trakarnsanga et al. describes for the first time the generation of such a cell line [21]. The authors immortalized adult CD34+ HSC by transduction with an inducible HPV16-E6/E7 construct. Briefly, after isolating the CD34+ HSC from donor bone marrow stem cells, the cells were grown in a medium containing cytokines (EPO, Transferrin, SCF, IL-3) for stimulation of erythroid progenitors. The cells were then infected with lentiviral vectors expressing HPV16-E6/E7 under the control of doxycycline-inducible promoter. The cells were transferred to an expansion medium containing EPO, SCF, dexamethasone and doxycycline that induced the expression of HPV16-E6/E7. The immortalized erythroid cell line was called BEL-A. The cell line was then induced into erythroid differentiation. BEL-A RBC were morphologically, molecularly and biochemically identical to those generated by peripheral blood. 94.9% of the derived RBCs produced α and β globin chains as per adult RBC. BEL-A reticulocytes showed the formation of a contractile ring of F-actin and myosin IIb next to the site of enucleation, as seen in peripheral blood reticulocytes. Proteomic analyses confirmed that the BEL-A RBCs no longer expressed the viral HPV16-E6/E7 proteins. Functional analysis also confirmed proper binding of haemoglobin with oxygen and carbon dioxide. When injected into immunodeficient mice (NSG) there was no difference between the survival of BEL-A RBC and adult donor RBC [21]. The BEL-A cells showed a mature erythrocyte morphology 24 h post injection and survived over the evaluation period (9 days). Further studies will be necessary to evaluate the safety and non-immunogenic nature of BEL-A RBC before starting a clinical trial.

In summary, this study confirmed that this immortalized erythroid cell line could be a reliable source of adult RBC. In contrast to the protocols developed for generation of RBC from peripheral and cord blood CD34+, HSC the method by Trakarnsanga et al. provides a stable not donor-limited source of RBC. In contrast to the protocols developed for generation of RBC from ESC and iPSC, the method by Trakarnsanga et al. generated RBC that were functionally identical to adult donor RBCs.

## Conclusion

The data reviewed indicate the superiority of such a cell line over ESC and iPSC as source of RBCs. Bone marrow, peripheral and cord blood have been considered not economically viable sources for RBC production because of the limited expansion of the progenitor cells and for the strict dependence of the protocol on cytokines and feeder layers. Trakarnsanga et al. present an interesting alternative and permanent source of RBCs able to produce terminally differentiated and functional RBC.

Would it be possible to scale-up this method in order to meet the requirements of industrial GMP production? Trakarnsanga et al. attempted to grow the cells in large spinner flasks and they did not observe issues with proliferation and differentiation however the manufacture of a proper bioreactor for RBC production and the replacement of the culture media with animal free GMP grade products was beyond the scope of the study. The use of viral proteins for the generation of the cell line is unlikely to raise a risk for clinical use: the expression of these proteins was lost before terminal differentiation and the mature RBC are enucleated and therefore do not carry any genetic material.

Notably Trakarnsanga et al. confirmed that the blood group corresponded to that of the bone marrow donor from which the cells were created. This means that with such a methodology it would be possible to generate RBCs of any blood group. This is particularly important for patients with rare blood groups.

## Abbreviations

CFU: Colony Forming Unit
EPO: Erythropoietin
ESC: Embryonic Stem Cells
G-CSF: Granulocyte-Colony Stimulating Factor
GMP: Good Manufacture Practice
HSC: Haematopoietic Stem Cells
IL3: Interleukin 3
iPSC: induced Pluripotent Stem Cells
RBC: Red Blood Cells
SCF: Stem Cell Factor
WHO: World Health Organisation
